# Interplay Between Reading and Writing Under Different Teaching Models: A Study Based on Chinese Learning by China’s Ethnic Minorities

**DOI:** 10.3389/fpsyg.2020.02150

**Published:** 2020-09-04

**Authors:** Ying Zhang, Hengli Peng, Yufang Bian

**Affiliations:** ^1^Collaborative Innovation Center of Assessment Toward Basic Education Quality, Beijing Normal University, Beijing, China; ^2^Faculty of Linguistic Sciences, Beijing Language and Culture University, Beijing, China

**Keywords:** reading, writing, teaching model, Chinese learning, gender difference

## Abstract

**Introduction:**

The relationship between reading and writing has been comprehensively explored from different perspectives. The following three theories and hypothesis could elucidate the relationship: reading→writing, writing→reading; and reading↔writing. In China, the teaching models of school influence the Chinese language learning of students in ethnic minority areas. Although language teaching can take various forms, this study selects two teaching models (S1: the traditional teaching model; S2: the complete Chinese teaching model) that can broadly represent Chinese minority schools. Primarily, this study aims to investigate the impact of different teaching models on the interplay between Chinese reading and writing ability of China’s minority students. Second, this study aims to explore gender differences in the relationship between reading and writing in two different teaching models.

**Methods:**

As the cross-lagged model is suitable for a longitudinal study of the data collected from multiple time waves and explore the causal relationship between variables. We enrolled 3869 Chinese ethnic minority fourth- to sixth- grade students from 126 schools and collected data for three waves. This study mainly achieves the two aims mentioned above through the cross-lagged design.

**Results:**

Results reveal that: (1) the complete Chinese teaching model is more effective than the mixed teaching model in stimulating the interaction relationship between reading and writing; (2) in the mixed teaching model, boys did not exhibit a significant effect of reading on writing, but only the effect of writing on reading, whereas girls exhibited the interaction between reading and writing; in the complete teaching model, there are gender differences in the relationship between reading and writing, however, with the development of time, the interaction between boys and girls in reading and writing becomes more robust, demonstrating that similar development trend in boys’ and girls’ interaction between reading and writing.

**Conclusion:**

The implication of these results is that: (1) the interactive relationship between reading and writing is developed in both teaching models; (2) there are some gender differences in the relationship between reading and writing in each teaching model.

## Introduction

It is commonly acknowledged that the four abilities of language (speaking, listening, reading, and writing) are interrelated. To date, several studies have comprehensively explored those relationships, especially the relationship between the two literacy skills, reading and writing (Berninger et al., 2002; Shanahan, 2009). Reading is the ability to extract, interpret, and use information from a print or digital text (Jonathan et al., 2018). We use a broad range of cognitive skills and language knowledge resources while engaging in reading comprehension activities (Perfetti and Adlof, 2012; Geva and Ramírez, 2015). Typically, the processes of reading require the abilities or language resources: accurate word recognition (i.e., lexical access), syntactic knowledge of the language, text structure and organizational patterns, and formulation of major ideas from text processing (Jonathan et al., 2018). Regarding the development of writing ability, increasing standards in children’s writing is a current educational priority (Department for Education, 2012, 2013). Writers could be influenced by their formal schema, which denotes knowledge about ways in which text types or genres are structured (Jonathan et al., 2018). Hence, writing in combination with students’ life experience or under the guidance of certain familiar topics is more likely to effectively develop real writing ability.

The relationship between reading and writing has been comprehensively investigated from various perspectives (Chapelle et al., 2011). Indeed, some studies extensively explored the relationship between reading and writing (Berninger et al., 1994, 2002; Abbott et al., 2010; Berninger and Abbott, 2010). However, whether the reading development led to the writing development or whether the writing development preceded the reading development remains unclear. Of note, three theories and hypothesis could help explain the relationship (Shanahan and Lomax, 1988; Schoonen, 2018).

The first theory proposes that reading ability contributes to the development of writing ability, corroborating the idea that the development of reading ability precedes writing. Some meta-analysis studies revealed that students from fourth to twelfth grade experienced the massive impact of the reading-to-writing teaching model or intervention on average (Scammacca et al., 2007, 2015). The second theory highlights that writing ability increases the development of reading ability. Such perspective holds that the development of writing skills is adequate, as its development comprises the development of reading ability and, consequently, promotes the development of reading skills. In addition, studies have demonstrated that the writing-to-reading model better explains the learning of beginners (Schoonen, 2018). The third theory integrates the most reasonable statements of the first two schools, that is, reading and writing mutually facilitate each other in their development. Abbott et al. (2010) examined the development of reading and writing skills of two groups of primary school students from the first to seventh grade and found that the reading and writing level had a strong autoregression for the same reading or writing level in the previous year, but a weak regression to overlapping skills, that is, the effect of reading on writing level was weak, and so was the effect of writing on reading level.

The research targets of previous studies were relatively small children in the early stage development of their reading and writing skills because children in this stage had strong plasticity and opportunities to receive reading and writing teaching and guidance. The majority of studies shed light on language education and usually involved the efficacy of language curriculum design in augmenting reading and writing skills (Shanahan, 2009). The relationship between different language teaching models and the development and relationship of reading and writing has also been a hot topic for research and the key to driving language education practice over the years.

Nevertheless, the equivocality of the research has suggested that the impact of different teaching models on the development of student reading and writing skills merits further research (Hall and Burns, 2018). In the field of writing research, teaching must cater to their abilities to make students learn effectively (Kamps and Greenwood, 2005; Al Otaiba and Fuchs, 2006). Reportedly, integration of the enhancement in reading and writing skills with teaching could decrease the incidence of language learning difficulties (Wanzek et al., 2010). In reading education, numerous successful reading instruction and intervention practices suggest that providing clear instructions to senior students was the key to obtaining a positive reading effect (Edmonds et al., 2009). Furthermore, providing appropriate practical opportunities and receiving valuable feedback in the teaching process correlated with enhanced academic performance (Hattie and Timperley, 2007; Shute, 2008).

As China is a multi-ethnic country, learning Chinese by Chinese minority students differs from learning their native languages. To a certain extent, learning Chinese for them is like learning a second language altogether. Hence, it is worth exploring the interplay between reading and writing ability for Chinese minority students during the development of reading and writing skills in Chinese at school. In addition, we focused on the role of Chinese teaching models on the development of reading and writing skills in minority students. This study was primarily based on the education project designed to teach Chinese to Chinese minority students and promote minority students to learn Chinese as a second language. We included the long-term and overall planning of reading and writing in teaching in the implementation process of the project to examine the effects of different bilingual teaching models on the development of students’ reading and writing skills in Chinese. Of note, fluent reading and writing training during Chinese teaching could promote the final formation and development of reading and writing abilities and the interplay between them. Moreover, the impact of long-term teaching model could be responsible for a unique interplay between Chinese reading and writing for Chinese minority students. Notably, discussion on this issue is conducive to determine the long-term effect model between teaching and language development in the teaching process and, subsequently, offer a basis to explore the impact of bilingual teaching models and some reference for the subsequent bilingual education planning in China’s minority areas.

Bilingual teaching is a vital component of the overall social, economic, cultural, and political environment. Different countries have different understandings of bilingual teaching and different models derived from practice (Baker, 2001, 2002). Typically, bilingual teaching denotes teaching activities with the mother tongue and second language as the media; however, its definition varies from country to country and from place to place. Internationally, bilingual teaching has had some broadly accepted definitions. The most authoritative definition of bilingual teaching, as defined in Longman Dictionary of Language Teaching and Applied Linguistics, is “the use of a second language or a foreign language in school for the teaching of content subjects” (1998). Owing to different cultural backgrounds of different countries, bilingual teaching differs in terms of concept, standard, objective, strategy, procedure, and models. Baker (1993) once categorized bilingual education models into 10 types; however, the popular models still widely used mainly include the following four types: transitional bilingual model; two-way bilingual model; immersion model; and maintenance bilingual education model.

The transitional bilingual model pertains to the education model in which the mother tongue is used partly or entirely after students enter the school, and later only the target language (second language) is adopted (Stern, 1999). The fundamental aim of the transitional bilingual model is to integrate minority students into the mainstream education (Baker, 2006). Specifically, in China, the transitional bilingual model aims to help students adapt to the second language classroom. Bilingual teachers teach children natural science, math, social science, and other subjects in students’ native language, while the second language is only used in the second language classroom, the and knowledge of other subjects can be used to communicate in the said classroom. Nevertheless, the theoretical advantages of this teaching model are challenging to be experienced in practice, mainly because students cannot quickly and accurately acquire the L2 words needed to communicate in other subjects, and, thus, it is difficult to realize a smooth transition to the second language classroom.

Cummins (2000) highlighted that the two-way bilingual model started in the United States and was gaining popularity. Baker (2001) argued that in a typical two-way bilingual model, students augmented their proficiency in both mother tongue and second language through learning, as well as used both languages equally in classroom teaching, that is, using the mother tongue and second language together. The two-way bilingual model accommodated two language groups together to promote the learning of a second language while sustaining the mother tongue. In addition, the two-way bilingual model promoted the academic accomplishment and language ability of both students whose language is used by the majority and the minority in the same classroom; this has triggered considerable interest in the United States. However, the focus should be on the limitation of using this model in China’s minority areas, that is, a limited number of minority schools with Chinese as a second language cannot fulfill the objective conditions of this bilingual environment and can only fulfill the requirements of Chinese teaching through the placement of corresponding teachers. Thus, the essential conditions of attending the same class for students of both Chinese nationality and ethnic minorities are not implemented yet in practice. Hence, it is challenging to put this bilingual teaching model into practice in China’s minority schools.

Longman Dictionary of Language Teaching and Applied Linguistics (1998) reported that the immersion bilingual model uses a single target language (second language) in teaching, rather than the child’s mother tongue. As a new type of second language education, the immersion bilingual model stemmed from the bilingual education model in Canada, particularly Quebec. After years of teaching practice in Canada, this bilingual model has been established to be effective and broadly recognized by the global bilingual society. Students learn scientific knowledge in a second language and receive an all-round development in the learning process. Indeed, students in China’s minority areas can learn Chinese using the Chinese immersion bilingual model through the placement of teachers whose mother tongue is Chinese, namely, adopting the complete Chinese teaching model examined in this study. Of note, teachers whose mother tongue is Chinese are qualified to teach Chinese in China’s minority areas. Besides using the unified Chinese textbook in the classroom, they mostly provide instructions and feedback to students in Chinese.

Baker (2002) reported that the essence of maintaining bilingual model is to educate minority students using both the minority and majority languages (second language). Notably, the maintenance bilingual model is also called bilingual teaching oriented to the traditional language of minority students. In this teaching model, the minority language is the dominant language in the majority of teaching, or, at least, takes up half of the course time (Dicker, 2003); this model is present in the traditional Chinese teaching model in schools in China’s minority areas, that is, the mixed teaching model, including the integration of the minority language and Chinese in this study. Chinese teachers in this model are primarily minority teachers who can speak Chinese in the minority areas; they have the qualifications needed to teach Chinese and have attained corresponding certifications. In addition, they usually use the unified textbook in Chinese teaching, while mainly relying on the minority language to provide guidance and feedback to minority students.

Previous research revealed that among students aged 9–10 years, girls scored higher than boys in writing ability (Babayiğit, 2015). Likewise, Midgette et al. (2008) found differences in the writing ability of the fifth- and eighth-grade students. The writing ability presents a consistency in terms of gender differences across different grades (Reynolds et al., 2015; Cordeiro et al., 2018). In addition, studies on reading abilities of primary school students revealed differences between boys and girls (Gao et al., 2019), and considerable literature showed that girls performed better in reading than boys (Voyer and Voyer, 2014; Nalipay et al., 2019). However, most studies focused on gender differences in writing or reading abilities. Some studies explored the relationship between writing and reading and their functions, with limited focus on gender differences, let alone exploring the impact of different teaching models on their relationship and gender differences. Yet, it is imperative to investigate the impact of different teaching models on the interplay between writing and reading abilities and their mutual promotion for students of different genders, which triggered our great concern in this study.

### This Study

As China is a multi-ethnic country, Chinese is one of the crucial subjects for students in China’s minority areas. In addition, Chinese is an essential instrument for minority students to secure better development and more opportunities in China in the future. Since 2002, “the Chinese Proficiency Test for Minorities in China” has been in practice to explore and adapt to the needs of Chinese teaching and testing in minority areas. The continuous maturity of the test has furthered our understanding of Chinese learning by minority students. Moreover, minority education has increasingly accentuated the need to adapt to the reading and writing development rules of minority students, as well as design teaching methods suitable for Chinese learning by minority students. Unlike learning their native languages, Chinese minority students learn Chinese as a second language, which is quite different from minority languages, as well as from alphabetic languages like English. Thus, proficient reading and writing ability is a crucial aspect of language ability to fulfill the needs of examination or actual communication. In addition, the teaching content needs to cater to the demand of students’ language development (Gao et al., 2019) to harness Chinese reading and writing skills.

In this study, among the teaching models described above, we adapted two models mainly for teaching Chinese in China’s minority areas—S1: mixed minority language-Chinese teaching model and S2: complete Chinese teaching model/Chinese immersion model. In addition, two different teaching models were adopted in the implementation of this study—mixed minority language-Chinese teaching model (S1) and complete Chinese model (S2). Besides, this study explores the roles of the models in the development of students’ reading and writing skills. Gender differences in both reading and writing have been frequently reported (Pajares and Valiante, 2001; McGeown et al., 2012; McGeown, 2013). Moreover, excellent reading and writing performance have been identified more closely with girls than boys (Millard, 1997; Marsh and Yeung, 1998; Guimond and Roussel, 2001; Pajares and Valiante, 2001; McGeown et al., 2012; McGeown, 2013). What are the differences in the way girls and boys develop their relationships between reading and writing? How do they develop differently in different teaching models? These questions provoked the interest of this study. This study further explores and examines the interplay between reading and writing abilities for boy and girl students under different teaching models. As the cross-lagged model facilitated exploring the longitudinal relationship between different variables across time, this study adopts it to examine the collected data, and then discover the development of writing and reading abilities for students of different genders and their interaction under different teaching models.

As overall planning in this study project, we attempt to compare the impact of different teaching models in teaching practice. To further examine the effect of each teaching model, we also examine gender differences using a 3-year longitudinal design to explore the relationship between reading and writing and examine whether this relationship depends on gender under each teaching model. This study could provide a basis for a comprehensive exploration of future similar studies and some reference for the choice of teaching models in the future.

## Materials and Methods

### Procedure

**FIGURE 1:**
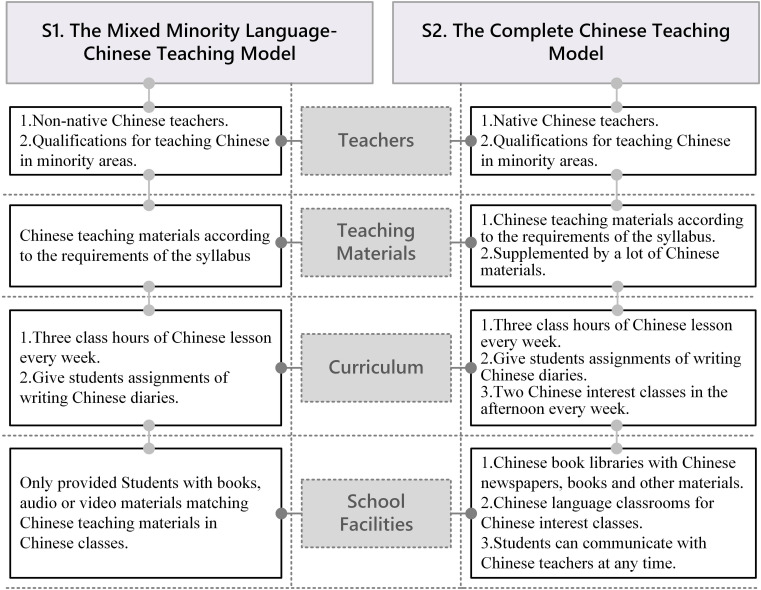
Brief introduction of two different Chinese teaching models in this study [i.e., The Mixed Minority Language-Chinese Model (S1) and The Complete Chinese Teaching Model (S2)].

Since the implementation of this study, we randomly selected different schools in Xinjiang ethnic minority areas for teaching design and arrangement of textbook use. We matched schools in terms of school socioeconomic status, facilities, teacher training, and Chinese textbook selection. We executed the teaching intervention in peer groups with the implementation of different teaching models in these peer groups. Hence, we established two groups differing only in the teaching model—S1: mixed minority language-Chinese group (traditional bilingual teaching group) and S2: complete Chinese teaching group (Chinese immersion teaching group). Teachers’ teaching methods were provided according to the research requirement and textbook use. In addition, differences in the impact of textbooks and teaching on students constituted different impacts of two teaching models in this study. Besides, teacher training for each group was provided regularly per the requirements of textbook and corresponding syllabus. Teachers in the S1 and S2 groups used the same Chinese Textbook (Fang, 2004). The major differences in Chinese teaching methods between these two groups are as follows (Figure 1): (i) *Composition of teachers*. Teachers in the S1 group were primarily comprised non-native Chinese teachers who were qualified for teaching Chinese in minority areas, whereas teachers in the S2 group mainly included native Chinese teachers who were qualified for teaching Chinese in minority areas. (ii) *Teaching materials*. The S1 group mostly used Chinese teaching materials in the teaching process to guide students to understand and master the teaching materials per the requirements of the syllabus, whereas the S2 group mainly relied on Chinese teaching materials, supplemented by plenty of Chinese materials, to guide students to understand and master the contents of teaching materials and Chinese materials per the syllabus and the content of teacher training, and used Chinese to guide students at ordinary times. (iii) *Arrangement of the Chinese course/Curriculum*. Schools assigned to the S1 and S2 groups participated in the uniform curriculum arrangement, respectively. Schools in the S1 group offered three class hours of Chinese lesson every week, whereas other courses in minority languages. Schools in the S2 group provided two Chinese interest classes in the afternoon every week besides three class hours of Chinese lesson every week. Students could choose to attend the interest classes for Chinese communication depending on their interests (all the lessons were offered in small class size, usually comprising 10–15 students). Native Chinese teachers performed various activities in the interest classes in Chinese (e.g., learning to sing Chinese songs and recite in Chinese). In training, both groups of Chinese teachers were encouraged to give students assignments of writing Chinese diaries, and every 2 weeks, students were required to write a composition regularly in class, for which, teachers provided written feedback. (iv) *School facilities*. Schools in the S1 group were primarily equipped with books, videos, and audio in the minority language traditionally, and only provided students with books, audio, or video materials matching Chinese teaching materials in Chinese classes. Schools in the S2 group were equipped with Chinese book libraries and Chinese language classrooms for Chinese interest classes in teaching buildings to provide students with an overall Chinese learning environment. Thus, students in the S2 group could read Chinese newspapers, books, and refer to Chinese materials at any time in the Chinese library located in their teaching building after class, as well as communicate with Chinese teachers at any time. Moreover, schools could use the school broadcast station to play Chinese programs for a certain length of time every day to provide students with a Chinese learning environment. Furthermore, teachers would play Chinese videos for students in class or in interest class.

The time for reading and writing test was 60 min in total. The chief examiners were uniformly trained by the education department in the minority areas, and the tests were invigilated uniformly. Finally, the objective questions were uniformly graded by the computer, and the subjective questions were graded by scorers who were engaged in Chinese examination for Chinese ethnic minorities for, at least, 3 years and received the uniform test and training.

### Participants

In this study, the data were primarily obtained from the stage data (2011−2013) of a longitudinal tracking project called “Studies on Chinese Communicative Competence Standards and Assessment System,” which was designed to take fourth to sixth graders (age: 9−12.5 years) learning Chinese in China’s minority areas as subjects (as this project study was a preliminary attempt, it only involved Xinjiang area where the Chinese Proficiency Test for Minorities in China has been in place for a relatively long period). Beijing Normal University and Beijing Language and Culture University and other units designed and implemented this study. As Chinese was a second language for students in minority areas, the main goal of a series of studies related to this project was to elucidate the current situation and regularity of Chinese learning for students in minority areas in China and offer better methods for future Chinese learning. Of note, this study was one of the series of studies in this project. The students participating in this study, as well as their schools, were subject to research follow-up and educational monitoring between 2011 and 2013, and relevant data were collected. Notably, the study participants involved in the span of 3 years in this study were all from the same batch. A total of 3869 primary school students (boys accounted for 46.7% of the total) from 126 schools participated in this study.

All the subjects were in the same grade and categorized into two groups (S1: mixed minority language-Chinese teaching method; S2: complete Chinese teaching method) based on the teaching methods (a total of 4803 students participated in the study). Students in both groups learned Chinese under two different teaching models and, finally, received the same test at the same time. Subjects started to engage in the study in the fourth grade, and tests were conducted for 3 consecutive years during the project research. Specifically, the first test time (T1) was the autumn semester in the fourth grade, the second test time (T2) was precisely at the same time in the second year, that is, the autumn semester in the fifth grade, for the same batch of subjects, and the same for the third test time (T3). A total of 4541 students participated in the first test, and some of them failed to participate in the second test because of transfer to another school, personal leave, sick leave, or other reasons. A total of 4201 students participated in the second test (with a loss rate of 7.49%) and 4128 students in the third test (with a loss rate of 9.09% compared with the first test). The analyses of the lost subjects, χ^2^ test, and variance analysis revealed no significant difference between the subjects participating in the second and third tests and the lost subjects in terms of gender ($x_{2}^{2}$ = 2.41, *p* = 0.19; $\chi_{1}^{2}$ = 2.33, *p* = 0.17), age [*F*_*1*_(1, 4538) = 1.03, *p* = 0.30; *F*_*2*_(1, 4538) = 1.34, *p* = 0.28], reading [*F*_1_(1, 4538) = 0.94, *p* = 0.45; *F*_*2*_(1, 4538) = 1.16, *p* = 0.31], and writing [*F*_*1*_(1, 4538) = 1.05, *p* = 0.44; *F*_*2*_(1, 4538) = 1.34, *p* = 0.28] in the first test, suggesting that sample loss was random. Overall, 3869 subjects participated in all the three tests, of whom, 43.8% were boys.

All school principals, students, and their guardians participating in this study provided a signed letter of consent to voluntarily participate in this study before the commencement of this study. We focused on assessing the impact of different bilingual teaching models implemented by schools to enhance students’ reading and writing abilities during the study process.

### Measures

The following measurement tools in this study were from the Collaborative Innovation Center of Assessment toward Basic Education Quality and the Faculty of Linguistic Sciences (FLS); these were mainly developed and used by the Beijing Normal University and Beijing Language and Culture University. This study aims to examine the language development of minority students and the relationship between individual development and educational factors in school. Of note, FLS, Beijing Language and Culture University [mainly accountable for the research and development of the Chinese Proficiency Test for Minorities in China (MHK) in ethnic minority areas] compiled numerous questionnaires on the standardized language test implemented in minority areas, which had been established to have both good reliability and validity (Peng, 2005).

#### School Questionnaire

The principals had to complete several multiple-choice questions related to the school environment of Chinese learning, including “Are the textbooks used in your school the uniformly compiled edition or self-selected ones?” (uniformly compiled by the Division of Ethnic Education, Ministry of Education/self-selected); “What is the current teaching model of the Chinese course in the bilingual class?”. Of note, questions of this part were used for grouping before the intervention of the teaching model so that schools participating in the experiment of two teaching models (S1: mixed minority language-Chinese/traditional bilingual teaching; S2: complete Chinese teaching group/Chinese immersion teaching) were assigned to equivalent groups. Before the formal survey, students randomly assigned to the S1 and S2 groups were given an initial test (i.e., reading and writing test for the fourth graders). However, no statistically significant difference was observed in terms of reading and writing scores between both groups of students under two teaching models, that is, the differences in subsequent research were mainly caused by differences in teaching models.

#### Reading Evaluation

The scores in this part were obtained from the read scores of the final-term Chinese examination (Yuan and Peng, 2014). There were 30 questions overall, of which, Questions 1−10 focused on language understanding in the discourse from the perspective of vocabulary knowledge, syntax, and comprehension. (1) Vocabulary knowledge examined in the first part signifies the knowledge pertaining to lexical meaning used by an individual to understand others’ speech and thoughts, and interpret the reading text (Moats, 2005), which is primarily tested using lexical definition and interpretation provided by students. The lexical quality hypothesis proposes that the quality of reading comprehension correlates with the quality of lexical knowledge (Perfetti, 2007). Thus, when studying the relationship between reading and writing development, vocabulary knowledge was placed in the first part of our study to test students’ reading level. In the test, we tested students’ interpretation of the lexical items appearing in the examination question (Li et al., 2009), and students’ vocabulary knowledge was examined by synonymous substitution or interpretation of a lexical item in a sentence in the form of multiple choices. The lexical items in the multiple-choice questions were subject to the Chinese syllabus. A total of 10 questions were set up in the study, with 1 point scored for each question, 0 point for an error or no answer. The highest score was 10 points and the lowest 0 point. A higher score suggested a higher quality for vocabulary knowledge. [For example, “with the joint efforts of the whole class, we can surely complete the task. A. fixedly; B. regularly; C. definitely; D. decidedly.” Making a correct choice warrants accurate understanding of the meaning of a word in a sentence. Another example is, “Unlike my sister, I am not interested in drawing. This meaning of the sentence is: A. I love painting; B. My sister can’t draw; C. My sister and I often draw; D. My sister likes drawing.” The difficulty in understanding this sentence lies in grasping the meaning of the negation word to choose from similar words a word that is most appropriate for the sentence; this question item tested students’ understanding of the meaning of a negation word in the context of a sentence]. (2) The second part examined syntax knowledge and mainly examined students’ use of words in sentences; this part included 10 questions, with 1 point for each correct answer, and 0 point for a wrong answer or no answer. The highest score was 10 points and the lowest 0 point. A higher score implied a higher syntactical level. In addition, this part focused on the students’ flexible application of vocabulary and grammar in a sentence [For example, “Each of us should carefully ________ the traffic rules.” As “
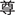
” in the option “
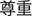
” (respect), and “
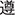
” in the option “
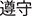
” share the same pronunciation and similar word form, this question examined students’ ability to distinguish words of the same pronunciation and similar forms. Another example, “Mom ________ herself very beautiful today. A. clothes; B. dressed up; C. expressed; D. image.” This question primarily examined students’ choice of words by considering its meaning expressed in the whole sentence, including choosing whether a verb or a noun, collocation of words, and overall application of syntactic rule)]. (3) Questions 21−30 in the third part mostly focused on examining students’ understanding of passages. There were four passages in total, with each passage containing about 100–150 Chinese characters, and there were 2 or 3 questions for each passage. Students answered questions about the meaning of words and sentences or the general idea of the passage by reading it, with 1 point scored for each correct answer, and 0 point for a wrong answer or no answer. The highest score was 10 points, and the lowest 0 point. A higher score implied a higher level for the comprehension of the passage.

The difficulty level of examinations in 3 years was adjusted per the curriculum standards and syllabus of each year. In addition, reading scores for comprehension questions were processed according to the equivalence method to form a longitudinal scale, allowing for comparison among different ages (among them, 5 questions were same in the questionnaire for the fourth to sixth graders, used for the longitudinal equivalence). As there was one correct answer to each question, so “1” point was scored for a correct answer or “0” for a wrong answer or no answer. The total score for each part (vocabulary, syntax, and comprehension) was 10 points. A higher score suggested a better mastery and a better competence in this part, and *vice versa*. As the overall reading scores were measured and equalized, the 3-year scores were comparable. Cronbach’s α was 0.88, the internal consistency was 0.87–0.94 in the study.

#### Writing Evaluation

**FIGURE 2:**
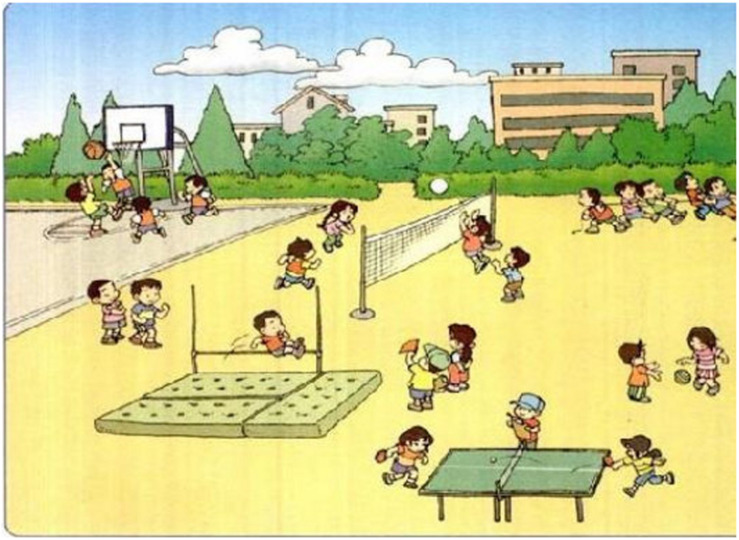
Situation prompt: On the playground, students, do sports, some…some…in high spirits (used in the second part of the writing test). (*Permissions have been obtained from Faculty of Linguistic Sciences*, *Beijing Language and Culture University*, *Beijing*, *China*).

The scores in this part primarily came from the assessment results of writing in the final-term Chinese examination. The assessment was obtained from the comprehensive evaluation of FLS. Notably, testing in the writing part was divided into spelling, the picture composition and composition. (1) the first part included 10 objective questions on spelling for Chinese phonetic alphabet (i.e., Chinese pinyin, Yuan and Peng, 2014). The objective questions were mainly set in accordance with the characteristics of Chinese, that is, each syllable of Chinese pinyin corresponded to a different Chinese character/word, and participants were required to write the correct Chinese character or word according to different contexts [e.g., “This performance is especially 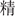 cai (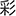) (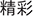 means *wonderful* in Chinese): I really want to watch another one.”] [an overall evaluation (0−6 points) was given by the scorer for this part]. A total of 10 questions were examined in the survey, in which 1 point was scored for a correct answer, and 0 point for a wrong answer or no answer. The highest score was 10 points and the lowest 0 point. A higher score implied a stronger ability in sentence spelling. The internal consistency coefficient of this part was 0.85–0.93 for the 3-year tests. (2) The following short composition was a short essay requiring students to write over 50 words. Students had to write a short essay corresponding to the situation shown in a picture and hinted through keywords (as shown in Figure 2) [an overall evaluation (0−6 points) was given by the scorer for this part]; (3) The second part was semi-guided writing (e.g., the semi-guided writing was “During our growth, we are grateful to many people, who may be our father, mother, teacher, or our classmates and friends. Write down what you want to say to him/her, and why you are thankful to him/her and your personal story. Please write a thank-you letter titled ‘A letter of thank you to XX’ according to the requirements of the letter format. Please complete the topic before writing. Write no less than 150 words in the genres other than poetry” [an overall evaluation (0−6 points) was given by the scorer for this part].

Based on the equivalence method, scores for objective questions were processed and converted into a longitudinal scale, enabling comparison among different ages (among them, 3 questions were same in the questionnaire for the fourth to sixth graders, used for the longitudinal equivalence). As there was one correct answer to each question, so “1” point was scored for a correct answer or “0” for a wrong answer or no answer. The total score for the first part was 10 points, and the second and third were writing (0–6 points). A higher score implied a better mastery in this part, and a lower score a poorer competence. As the overall writing scores were measured and equalized, the 3-year scores were comparable. Cronbach’s α was 0.85, the internal consistency was 0.86–0.92 in the study.

### Statistical Analysis

We performed measurement equivalence processing on the 3-year data to render the reading and writing assessment results over the 3 years comparable in time. For convenience, we used an IRT model for vertical scaling. To develop a vertical scale, achievement growth between a pair of grades was defined as the change in scores over the content. We used BILOG 3.09 for the conversion of scale scores based on the subsequent reading and writing score comparison. We adopted a classic three-wave, cross-lagged panel design. We hypothesized that the development of reading and writing skills of Chinese minority students could facilitate each other in Chinese learning. Besides, different teaching models could further impact this type of mutual facilitation. We used Mplus8.0 for modeling and analysis of the cross-lagged panel structural model and SPSS19.0 for the primary analysis and processing of the data.

### Ethics Statement

In this study, the core variables were the students’ reading and writing scores over 3 years, which were graded by Chinese experts based on the reading and writing evaluation criteria, respectively. We surveyed students in the classroom at every wave of the timeline. The Institutional Review Committee, comprising the Collaborative Innovation Center of Assessment toward Basic Education Quality, Beijing Normal University and Faculty of Linguistic Sciences, Beijing Language and Culture University, approved all the questionnaires and procedures used in this study. In addition, written informed consent was obtained from all principals, students and their parents in this study.

## Results

### Descriptive Statistics

The multivariate analysis of variance (MANOVA) revealed that different class models [0 = parallel teaching model adopting the mixed minority language and Chinese (S1); 1 = complete Chinese teaching model (S2)] exerted a significant effect on reading at three waves [*F*(2,3866) = 74.81, *p* < 0.01, partial η^2^ = 0.41]. Then, the univariate analysis revealed that students in the complete Chinese teaching model exhibited a higher reading level in each time period. Moreover, MANOVA suggested that the class model exert a significant effect on writing at three waves of measurement [*F*(2,3866) = 42.46, *p* < 0.05, partial η^2^ = 0.27], and follow-up univariate analysis also suggested that the complete teaching model had higher levels of writing ability at each interval (Table 1).

**TABLE 1:** Mean score (*M*), standard deviation (*SD*) and MANOVA results.

TM	Variable	Evaluation	*M(SD)*			*F_(T1–T3)_*	$\eta_{p}^{2}$
			
			T1	T2	T3		
S1	Reading	*Vocabulary knowledge (0-10)*	4.01(0.97)	4.79(1.18)	6.68(1.98)		
		*Syntax (0-10)*	4.18(1.03)	5.27(1.19)	6.95(1.23)	74.81**	
		*Comprehension (0-10)*	4.88(1.22)	5.75(1.43)	7.27(1.35)		0.41
	Writing	*Sentence spelling (0-10)*	5.77(1.22)	6.04(1.31)	7.56(1.27)		
		*Short composition (0-6)*	2.94(1.15)	3.18(1.22)	4.33(1.19)		
		*Semi-guided Writing (0-6)*	2.06(1.13)	3.29(1.31)	4.51(1.28)		
S2	Reading	*Vocabulary knowledge (0-10)*	4.09(1.01)	4.96(1.92)	6.79(1.53)		
		*Syntax (0-10)*	4.30(1.19)	5.58(1.33)	7.05(1.35)		
		*Comprehension (0-10)*	4.84(1.58)	6.64(1.75)	9.07(1.46)	42.46*	0.27
	Writing	*Sentence spelling (0-10)*	6.12(1.21)	7.47(1.28)	8.13(1.19)		
		*Short composition (0-6)*	3.03(1.13)	3.99(1.08)	4.97(1.23)		
		*Semi-guided writing (0-6)*	2.29(1.56)	4.08(1.68)	5.77(1.49)		

TM, teaching model; S1, the mixed minority-Chinese teaching model; S2, the complete Chinese teaching model. T1 denotes the survey data in the first year; T2 denotes the survey data in the second year; and T3 denotes the survey data in the third year. *p < 0.05 and **p < 0.01.

### Correlation Analysis

In this study, we performed a correlation analysis of subjects’ reading and writing abilities and corresponding control variables in the three tests under two different teaching models (Table 2). We observed a significant correlation between students’ reading and writing, whether at the same time-point or at different time-points (*ps* < 0.01). Overall, the correlation coefficient between reading and writing at different time-points ranged 0.302–0.583.

**TABLE 2:** Correlation coefficient of all students, mean score (*M*) and standard deviation (*SD*) of boys and girls in different teaching models about reading and writing.

Variable	1	2	3	4	5	6
(1) Reading (T1)	1	0.443***	0.375***	0.444***	0.323***	0.324***
(2) Reading (T2)	0.583***	1	0.487***	0.375***	0.427***	0.404***
(3) Reading (T3)	0.527***	0.490***	1	0.327***	0.382***	0.378***
(4) Writing (T1)	0.410***	0.417***	0.361***	1	0.412***	0.421***
(5) Writing (T2)	0.302**	0.507***	0.370***	0.435***	1	0.437***
(6) Writing (T3)	0.305**	0.462***	0.332***	0.391***	0.415***	1
**S1**						
*Girls (M/SD)*						
M	13.13	15.90	21.07	7.79	11.28	14.54
SD	4.59	4.26	4.85	1.98	2.05	2.06
*Boys*						
M	13.01	15.72	20.33	7.98	7.71	10.80
SD	5.01	4.44	4.33	2.23	2.16	2.39
**S2**						
*Girls*						
M	13.47	18.35	24.07	8.49	8.82	15.65
SD	4.39	4.19	4.76	2.11	2.23	2.19
*Boys*						
M	12.99	16.01	21.95	8.27	7.32	13.29
SD	4.63	4.22	4.65	1.99	2.17	2.24

The table is divided into three parts, the first part of the table is the correlation coefficient of all the students, the correlation coefficient in the mixed minority language-Chinese teaching model (S1) is presented in the upper triangle, while the correlation coefficient of the complete Chinese teaching model (S2) is presented in the lower triangle. T1, the survey data in the first year; T2, the survey data in the second year; and T3, the survey data in the third year. **p < 0.01 and ***p < 0.001.

### Relationship Between Writing and Reading: Test Based on the Cross-Lagged Panel Structural Model

Before exploring the teaching model effects in the relationship between reading and writing, a series of nested CFAs were conducted to obtain evidence of measurement invariance across girls and boys. The measurement structure of latent factors was freely estimated across girls and boys in the unconstrained model, and the factors and factor loading patterns were constrained to be equal across two groups in the constrained model. Next, the *x*^2^ difference test was used to assess significant differences in the models across gender groups (Table 3). All *x*^2^ differences were significant for reading and writing, while other fit indices did not substantially decrease (ΔCFI < 0.01, ΔTLI < 0.01, ΔRMSEA < 0.005). These findings suggested that reading and writing display factorial invariance across teaching model groups.

**TABLE 3:** Goodness-of-fit of measurement invariance confirmatory structural equation model between S1 and S2 for reading and writing.

Variable	Model	χ^2^	*df*	CFI	TLI	RMSEA [90% CI]	Δχ^2^	Δ*df*	ΔCFI	ΔTLI	ΔRMSEA	*p*
Reading	U	72.554	48	0.984	0.976	0.047[0.032, 0.058]	2.697	6	+0.002	+0.003	−0.002	<0.001
	C	75.251	54	0.986	0.979	0.045[0.033, 0.057]						
Writing	U	98.495	48	0.974	0.961	0.051[0.039, 0.062]	4.674	6	+0.001	+0.003	+0.002	<0.001
	C	103.169	54	0.975	0.964	0.053[0.041, 0.061]						

*U (unconstrained) means the unconstrained model that measurement structure of latent factors is freely estimated between the mixed minority language-Chinese teaching model (S1) and the complete Chinese teaching model (S2), C (constrained) means the constrained model that the factors and factor loading patterns were constrained to be equal across two groups.*

We performed a comparative analysis of multiple groups to test the path difference between the two teaching models after controlled vocabulary knowledge and phonological awareness. (1) We established two groups of cross-lagged baseline models (primarily denoting reading and writing at three waves). All paths allowed free estimation according to the teaching model and attained a good fit (*x*^2^ = 1154.398, *df* = 630, *p* < 0.001, CFI = 0.97, TLI = 0.94, RMSEA = 0.030 with 90% CI = [0.015, 0.043]). (2) We established a constrained cross-lagged model, setting the structural load weight between writing and reading to be equal between paths corresponding to different teaching models, and enabled the measurement model to change freely at three waves (*x*^2^ = 1365.622, *df* = 557, *p* < 0.001, CFI = 0.92, TLI = 0.90, RMSEA = 0.036 with 90% CI = [0.029, 0.054]). (3) The χ^2^ difference test suggested that compared with the unconstrained model, the fitting degree of the constrained model decreased marginally (Δ$\chi_{\left(73\right)}^{2}$ = 211.224, *p* < 0.001), and other suitable indexes also worsened (ΔCFI > 0.02, ΔTLI > 0.02, ΔRMSEA > 0.005), suggesting that the constraint model was rejected, that is, the relationships between reading and writing skills varied with the teaching model.

**FIGURE 3:**
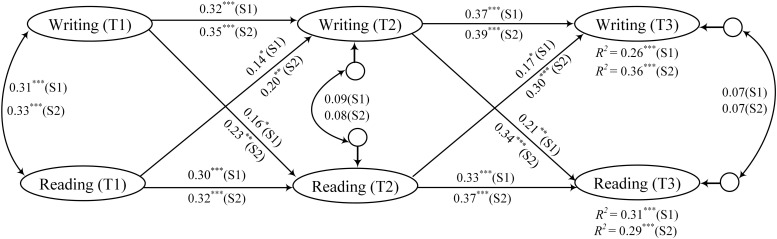
Standardized regression coefficients in cross-lagged panel structural model in the mixed minority language-Chinese teaching model (S1) and the complete Chinese teaching model (S2). ***p < 0.001; **p < 0.01; and *p < 0.05.

As shown in Figure 3, in the mixed minority language-Chinese teaching model, students’ writing and reading skills interacted with each other and progressed, mostly in that students’ writing facilitated the development of reading, while reading played a relatively weak role in enabling the development of writing. In the complete Chinese teaching model, students’ reading and writing mutually facilitated each other, exhibiting an even stronger promoting effect. Specifically, under the mixed minority language-Chinese teaching model (S1), reading at T1 and T2 could significantly predict reading at T2 and T3 (β_1_ = 0.30, *p*_1_ < 0.001; β_2_ = 0.33, *p*_2_ < 0.001), and writing at T1 and T2 could significantly predict writing at T2 and T3 (β_1_ = 0.32, *p*_1_ < 0.001; β_2_ = 0.37, *p*_2_ < 0.001). Reading at T1/T2 could significantly predict writing at T2/T3 (β_1_ = 0.14, *p*_1_ < 0.05; β_2_ = 0.17, *p*_2_ < 0.05), and writing at T1/T2 could significantly predict reading at T2/T3 (β_1_ = 0.16, *p*_1_ < 0.05; β_2_ = 0.21, *p*_2_ < 0.01). Under the complete Chinese teaching model (S2), reading at T1/T2 could significantly predict reading at T2/T3 (β_1_ = 0.32, *p*_1_ < 0.001; β_2_ = 0.37, *p*_2_ < 0.001), and writing at T1/T2 could significantly predict writing at T2/T3 (β_1_ = 0.35, *p*_1_ < 0.001; β_2_ = 0.39, *p*_2_ < 0.001). Reading at T1/T2 could significantly predict writing at T2/T3 (β_1_ = 0.20, *p*_1_ < 0.01; β_2_ = 0.30, *p*_2_ < 0.001), and writing at T1/T2 could significantly predict reading at T2/T3 (β_1_ = 0.23, *p*_1_ < 0.01; β_2_ = 0.34, *p*_2_ < 0.001).

### Gender Differences in the Relationship Between Reading and Writing Under Different Teaching Model Groups

We analyzed teaching activities performed in the two teaching models to understand the specific influence of each teaching model on the interplay between reading and writing for boy and girl students. Before examining the gender effects in the relationship between reading and writing, a series of nested CFAs were conducted to obtain evidence of measurement invariance across boys and girls. The measurement structure of latent factors were freely estimated across boys and girls in the unconstrained model, and the factors and factor loading patterns were constrained to be equal across two groups in the constrained model. Then, the *x*^2^ difference test was used to assess significant differences in the models across gender groups, and the results were shown at Table 4. The results suggested that under the S1/S2 teaching model, both reading and writing exhibited a factorial invariance across gender groups.

**TABLE 4:** Goodness-of-Fit of measurement invariance confirmatory structural equation model between boys and girls for reading and writing under each teaching model (S1 and S2).

TMM	Variable	Model	χ^2^	*df*	CFI	TLI	RMSEA [90% CI]	Δχ^2^	Δ*df*	ΔCFI	ΔTLI	ΔRMSEA	*p*
	Reading	U	84.374	48	0.967	0.961	0.046[0.040, 0.058]	3.475	6	−0.002	+0.002	+0.001	<0.001
**S1**		C	87.849	54	0.965	0.963	0.047[0.041, 0.058]						
	Writing	U	80.610	48	0.973	0.968	0.049[0.043, 0.061]	4.445	6	+0.002	−0.002	+0.001	<0.001
		C	85.055	54	0.975	0.966	0.050[0.043, 0.061]						
	Reading	U	99.930	48	0.956	0.941	0.041[0.033, 0.052]	2.035	6	−0.002	−0.003	−0.002	<0.001
**S2**		C	101.965	54	0.954	0.938	0.039[0.033, 0.049]						
	Writing	U	76.595	48	0.969	0.953	0.043[0.036, 0.057]	6.358	6	−0.001	+0.002	+0.001	<0.001
		C	82.953	54	0.968	0.955	0.044[0.036, 0.057]						

*TM, teaching model (including S1, the mixed minority language-Chinese teaching model; S2, the complete Chinese teaching model); U (unconstrained), the unconstrained model that measurement structure of latent factors is freely estimated between boys and girls; C (constrained), the constrained model that the factors and factor loading patterns were constrained to be equal across two groups.*

In this study, multigroup comparisons were conducted to test differences in paths across gender in the S1 and S2 teaching models separately. We established two groups of cross-lagged baseline models (primarily denoting reading and writing at three waves). (1) A two-group cross-lagged model as the baseline model (including reading and writing over the three waves), in which all of the paths were left free to vary by gender, achieved reasonable fit (*x*^2^ = 1889.253, *df*_(S1)_ = 630, *p*_(S1)_ < 0.001, CFI_(S1)_ = 0.95, TLI_(S1)_ = 0.93, RMSEA_(S1)_ = 0.041 with 90% CI = [0.034–0.052]; *x*^2^ = 1902.253, *df*_(S2)_ = 630, *p*_(S2)_ < 0.001, CFI_(S2)_ = 0.96, TLI_(S2)_ = 0.95, RMSEA_(S2)_ = 0.038 with 90% CI = [0.032–0.050]). (2) A constrained cross-lagged model was conducted, in which structural weights between reading and writing were set to be equal to corresponding paths across gender, and the measure model were left free to vary across three waves (*x*^2^ = 964.908, *df*_(S1)_ = 553, *p*_(S1)_ < 0.001, CFI_(S1)_ = 0.92, TLI_(S1)_ = 0.90, RMSEA_(S1)_ = 0.052 with 90% CI = [0.045–0.063]; *x*^2^ = 982.473, *df*_(S2)_ = 630, *p*_(S2)_ < 0.001, CFI_(S2)_ = 0.93, TLI_(S2)_ = 0.90, RMSEA_(S2)_ = 0.042 with 90% CI = [0.036–0.054]). (3) The χ^2^ difference test suggested that compared with the unconstrained model, the fitting degree of the constrained model decreased marginally (Δ$\chi_{\left(s\,1\right)}^{2}$ = 924.345, *p*_(S1)_ < 0.001; Δ$\chi_{\left(s\,2\right)}^{2}$ = 919.780, *p*_(S2)_ < 0.001), and other suitable indexes also worsened (ΔCFI_(S1)_ > 0.02, ΔTLI_(S1)_ > 0.02, ΔRMSEA_(S1)_ > 0.005; ΔCFI_(S2)_ > 0.02, ΔTLI_(S2)_ > 0.02, ΔRMSEA_(S2)_ > 0.005), suggesting that the two models were rejected, that is, the relationship between reading and writing skills varied by gender under S1 (and S2) teaching model.

**FIGURE 4:**
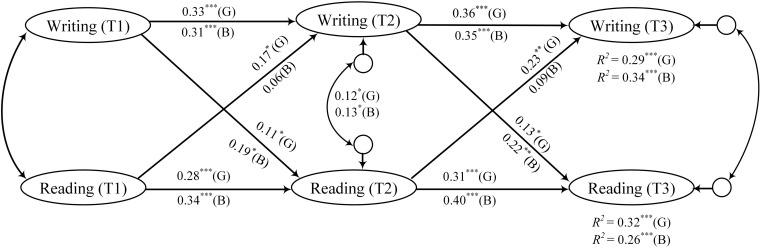
Mixed minority language-Chinese teaching model. Standardized regression coefficients in cross-lagged panel structural model in boys (B) and girls (G) under the mixed minority language-Chinese teaching model. ***p < 0.001; **p < 0.01; and *p < 0.05.

In the mixed minority language-Chinese teaching model (Figure 4), the results revealed that girls’ reading at T1/T2 could significantly predict their reading at T2/T3 (β_1_ = 0.28, *p*_1_ < 0.001; β_2_ = 0.31, *p*_2_ < 0.001). Similarly, boys’ reading at T1/T2 could also significantly predict their reading at T2/T3(β_1_ = 0.34, *p*_1_ < 0.001; β_2_ = 0.40, *p*_2_ < 0.001). Girls’ writing at T1/T2 could significantly predict their writing T2/T3 (β_1_ = 0.33, *p*_1_ < 0.001; β_2_ = 0.36, *p*_2_ < 0.001). Likewise, boys’ writing at T1 and T2 could significantly predict T2/T3 writing (β_1_ = 0.31, *p*_1_ < 0.01; β_2_ = 0.35, *p*_2_ < 0.001). Girls’ reading at T1/T2 could significantly predict their writing at T2/T3 (β_1_ = 0.17, *p*_1_ < 0.05; β_2_ = 0.23, *p*_2_ < 0.01). However, boys’ reading at T1/T2 fails to significantly predict their writing at T2/T3 (*ps* > 0.05). Girls’ writing at T1/T2 could significantly predict their reading at T2/T3 (β_1_ = 0.11, *p*_1_ < 0.05; β_2_ = 0.13, *p*_2_ < 0.05). In addition, boys’ writing at T1/T2 could significantly predict their reading at T2/T3 (β_1_ = 0.19, *p*_1_ < 0.05; β_2_ = 0.22, *p*_2_ < 0.01). The model-fitting results revealed that in the interaction relationship between reading and writing embodied in the mixed minority language-Chinese teaching model, boys’ reading exerted little effect on writing, and this relationship was not changed over time. Their writing could promote reading somewhat; this effect was marginally strengthened with development. The interplay trend between the reading and writing development under this teaching model was significant for girls, and this effect was marginally strengthened with development. Briefly, in the traditional mixed minority language-Chinese teaching model, students’ reading and writing relied more on their own accumulation and promotion, and the interaction between the two was not prominent. As teachers in traditional teaching used their native language to guide students, and students mostly relied on test-taking skills to enhance their Chinese grades, it was challenging for students to really improve their reading and writing abilities. Hence, it was difficult for students to promote their learning effects and achieve mutual promotion between reading and writing.

**FIGURE 5:**
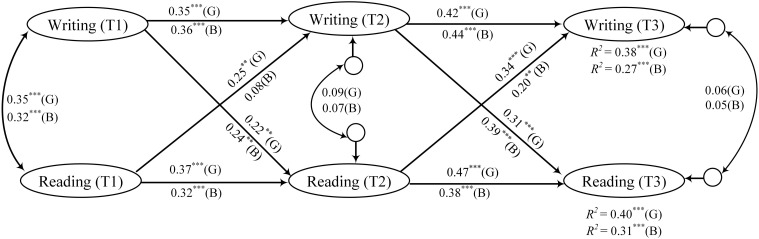
Complete Chinese teaching model. Standardized regression coefficients in cross-lagged panel structural model in boys (B) and girls (G) under the complete Chinese teaching model. ***p < 0.001 and **p < 0.01.

As shown in Figure 5, in the complete Chinese teaching model, the results revealed that girls’ reading at T1/T2 could significantly predict their reading at T2/T3 (β_1_ = 0.37, *p*_1_ < 0.001; β_2_ = 0.47, *p*_2_ < 0.001). Similarly, boys’ reading at T1/T2 could also significantly predict their reading at T2/T3 (β_1_ = 0.32, *p*_1_ < 0.001; β_2_ = 0.38, *p*_2_ < 0.001). Girls’ writing at T1/T2 could significantly predict their writing at T2/T3 (β_1_ = 0.35, *p*_1_ < 0.001; β_2_ = 0.42, *p*_2_ < 0.001). Likewise, boys’ writing at T1/T2 could significantly predict their writing at T2/T3 (β_1_ = 0.36, *p*_1_ < 0.001; β_2_ = 0.44, *p*_2_ < 0.001). Girls’ reading at T1/T2 could significantly predict their writing at T2/T3 (β_1_ = 0.25, *p*_1_ < 0.01; β_2_ = 0.34, *p*_2_ < 0.001). However, boys’ reading at T1/T2 failed to significantly predict their writing at T2/T3 (β = 0.08, *p* > 0.05), while boys’ reading at T2 could significantly predict T3 writing (β = 0.20, *p* < 0.01). Girls’ writing at T1/T2 could significantly predict their reading at T2/T3 (β_1_ = 0.22, *p*_1_ < 0.01; β_2_ = 0.31, *p*_2_ < 0.001). Likewise, boys’ reading at T1/T2 could also significantly predict their reading at T2/T3 (β_1_ = 0.24, *p*_1_ < 0.01; β_2_ = 0.39, *p*_2_ < 0.001). From the perspective of the model fitting results, girls’ reading exhibited an increasingly stronger promotion on writing with the implementation of the complete Chinese teaching model. In addition, girls’ writing exhibited an increasingly promoting effect on reading. Moreover, the interplay between girls’ writing and reading abilities exerted a significant impact at various stages. Although boys also exhibited a trend that writing exerted an increasingly stronger impact on reading than the other way around, at first, boys’ reading did not have a significant impact on writing, and it is only in the second year that the trend that reading promoted writing began to emerge, which shows the promoting effect of teaching model. We observed a similar trend between boys and girls in the way that writing affected reading. Under the complete Chinese teaching model, a mutual interplay was noted between reading and writing to varying degrees for boys and girls.

## Discussion

Research on the application of certain educational models or intervention means to efficiently promote the development of students’ reading and writing skills has proliferated considerably (McKenna and Stahl, 2018; Treiman, 2018). In this study, we adopted a 3-year longitudinal design to elucidate the role played by different teaching models in promoting the relationship between reading and writing skills from the developmental perspective. The findings revealed that regardless of the teaching model adopted in the process of China’s minority students learning Chinese, reading and writing could facilitate the development of each other to varying degrees. Schoonen (2018) argued that both reading and writing needed language learners to display a certain fluency in acquiring language knowledge resources. Furthermore, this study revealed that the relationship between reading and writing differed to varying degrees because of different teaching models. This study also reported gender differences in the interplay between Chinese reading and writing under different teaching models. All these provide some reference for the subsequent teaching design and method selection to implement Chinese teaching in minority areas in China.

First, this study constructs an overall model to explore the promotion of the S1 and S2 teaching models on the development of Chinese reading and writing ability, respectively. Under the minority language-Chinese teaching model (S1), writing and reading skills mutually facilitated each other, and the relationship between the two was relatively weak. Teachers who used this teaching model were from the said ethnic minority, with the minority language as their native language; their guidance on Chinese provided to students was mostly realized through their native language, and such guidance was depicted more in writing and examination skills. In Chinese teaching, besides Chinese textbooks, teachers did not have adequate Chinese materials to help children read sufficiently, and teachers whose native language was not Chinese were unable to provide students with insightful guidance in Chinese. Under the S1 teaching model, teachers often left assignment such as writing diaries in Chinese. The form of assignment, that is, writing in limited Chinese, was more familiar to students, and students often mobilized Chinese words already known to them in writing. In a writing task, students must complete a reading task and use their internal memory and language resources to complete the task (Hayes, 1996, 2012), under the S1 teaching model, students could also engage in Chinese reading in the assigned Chinese writing task, students had to further mobilize their reading ability (Flower and Hayes, 1981; Hayes, 1996), thus, students’ high writing ability encouraged students to improve their reading ability. However, although reading needs induced by Chinese writing and Chinese accumulation could facilitate the development of Chinese reading to a certain degree, this type of facilitation was limited because of insufficient materials, which was reflected in the path coefficient of model fitting.

For the complete Chinese teaching model (S2), writing and reading ability displayed more robust mutual facilitation, and the mutual interplay, that is, writing ability on reading ability, and *vice versa*, was nearly the same. Some previous studies proposed that reading ability favors the writing process and writing output (McCutchen, 2000). Considerable Chinese language materials and language teachers provide significant guided communication, enabling students obtain higher quality of Chinese reading at ordinary times. Students have to do daily Chinese writing exercises (e.g., Chinese diary), which would be useful at ordinary times reading material content in the application of Chinese writing. Reading and feedback occasionally play a prominent role in reading comprehension and writing revision, including weighing the role of vocabulary and characteristics of the writing style, and grasping of sentence structure (Deane et al., 2008). Usually, students must write according to the written materials they read and must create a mental model for the writing task (Nicola’s-Conesa, 2012), which itself could warrant careful reading of the instructions or the original materials. To better complete the writing task, students mobilize their internal mental model to read articles till the time of writing (Hayes et al., 1987).

In addition, we found gender differences in the developmental relationship between Chinese reading and writing under each teaching model. Several studies on reading and writing have reported differences in reading and writing abilities between boys and girls. Though most studies suggested that girls perform better in reading and writing than boys (Reynolds et al., 2015; Cordeiro et al., 2018), some studies reported that girls do not always enjoy advantages in terms of reading or writing ability compared with boys (Jewell and Malecki, 2005; Beard and Burrell, 2010; Scheiber et al., 2015). Despite divergent conclusions drawn for gender differences in reading and writing, the relationship between reading and writing is more complicated. This study focused on the effect of different teaching models on the relationship between reading and writing. Specifically, under the S1 teaching model, boys’ reading ability only affected its own development, and its development hardly contribute to the development of writing ability. However, the development of boys’ writing ability markedly facilitated the development of reading ability. Similarly, the development of girls’ reading ability exerted a significant impact on the development of their writing ability. Under the S2 teaching model, the development of reading and writing for boys and girls exhibited mutual facilitation. Obviously, under the teaching model with abundant Chinese resources and in-depth guidance for Chinese learning, both boys and girls could achieve integrated development of reading and writing, thereby exhibiting a more significant interplay between each other.

This study has a certain theoretical significance. Different theories have been used to elucidate the interplay between reading and writing. For example, a one-way interaction (i.e., reading influences writing, or *vice versa*) could exist between reading ability and writing ability, or a two-way interaction (Shanahan and Lomax, 1988; Schoonen, 2018). In this study, we set up different teaching models to compare the interplay between the development of reading ability and that of writing ability under different teaching models. In the process of Chinese minority students learning Chinese, the traditional S2 teaching model could better promote the interaction between reading and writing compared with the S1 teaching model. Thus, we found gender differences between boys and girls. In addition, a mutual promotion was present between reading and writing for girls under these two teaching models. However, boys’ reading and writing could facilitate each other to a certain extent under the S2 teaching model. One-way facilitation, that is, writing facilitating reading, was observed in the S1 teaching model; that is, reading did not significantly impact writing in this model, suggesting that in the future research on the relationship between reading and writing, we should not only take the overall development relationship between reading and writing into account but also comprehensively weigh teaching models and individual factors (such as gender in this study) that can promote the development of reading and writing to provide a detailed explanation. Moreover, this study has some practical significance. Our study demonstrates that the interplay between reading and writing is less significant for Chinese minority students learning Chinese under the traditional S1 teaching model compared with the S2 teaching model, and such differences are more viewed from the perspective of gender differences. It is crucial to provide students with sufficient Chinese reading materials, reading and writing guidance from native Chinese teachers, and more frequent activities organized for small Chinese interest classes. This study can inspire teachers and educational decision-makers to take more extensive measures, such as appropriately increasing teaching input based on the original teaching input, helping students gradually improve their reading and writing abilities by providing more reading materials and strengthening the training of native language teachers, and improving the interplay between reading and writing.

### Limitations

This study has some limitations worth acknowledging. First, the study participants were students from the minority groups in Xinjiang, China, which was favorable to control the influence of students’ native language. However, as China is a multi-ethnic country, the diversity of background languages should be considered when promoting a complete Chinese teaching model in other minority areas in China. Second, owing to the limitation of data collection and study design, we could not determine whether the differences in other aspects of the school environment are accountable for differences in reading and writing skills under different teaching models. Thus, a comprehensive influence mechanism of teaching models remains to be further examined. Finally, although this study aimed to investigate reading and writing skills, only a limited number of crucial variables were included in this study. Hence, future research should further control these variables or elucidate the impact of other variables on reading and writing skills.

## Conclusion

This study establishes an interactive relationship between reading and writing in both teaching models. The complete Chinese teaching model is more effective than the mixed teaching model in promoting the interaction relationship between reading and writing. In the mixed teaching model, boys do not show a significant effect of reading on writing, but only the effect of writing on reading, whereas girls exhibit the interaction between reading and writing. In the complete teaching model, there are gender differences in the relationship between reading and writing, however, with the development of time, the interaction between boys and girls in reading and writing becomes more robust, demonstrating that similar development trend in boys’ and girls’ interaction between reading and writing.

## Data Availability Statement

The raw data supporting the conclusions of this article will be made available by the authors, without undue reservation.

## Ethics Statement

The Institutional Review Committee, comprising the Collaborative Innovation Center of Assessment toward Basic Education Quality, Beijing Normal University and Faculty of Linguistic Sciences, Beijing Language and Culture University, approved all the questionnaires and procedures used in this study. In addition, written informed consent was obtained from all principals, students and their parents in this study.

## Author Contributions

YZ designed and executed the study, analyzed the data, and wrote the manuscript. HP collaborated with editing of the manuscript. YB collaborated with the design of the study. All authors contributed to the article and approved the submitted version.

## Conflict of Interest

The authors declare that the research was conducted in the absence of any commercial or financial relationships that could be construed as a potential conflict of interest.
